# Cerebral amyloid angiopathy decades after red blood cell transfusions: a report of two cases from a prospective cohort

**DOI:** 10.1111/ene.16277

**Published:** 2024-03-18

**Authors:** K. Kaushik, M. J. H. Wermer, E. S. van Etten

**Affiliations:** ^1^ Department of Neurology Leiden University Medical Centre (LUMC) Leiden The Netherlands; ^2^ Department of Neurology University Medical Centre Groningen Groningen The Netherlands

**Keywords:** blood transfusion, cerebral amyloid angiopathy, iatrogenic CAA, intracerebral hemorrhage, prion, RBC transfusion, red blood cell, stroke, transmission

## Abstract

**Background and purpose:**

Patients who underwent red blood cell (RBC) transfusion from donors who later developed multiple spontaneous intracerebral hemorrhages (ICHs) have recently been identified to have increased risk of ICH themselves. This increased risk of ICH was hypothesized to be related to iatrogenic cerebral amyloid angiopathy (iCAA) transmission. Two cases are presented who had RBC transfusion as an infant and presented with CAA at a relatively young age decades later.

**Method:**

Cases were identified by prospectively asking all patients at our CAA outpatient clinic (November 2023 to January 2024) about a medical history with RBC transfusion or history with a high likelihood for RBC transfusion (e.g., hemolytic disease, trauma with massive hemorrhage). Eligible patients were all diagnosed with CAA, CAA with concomitant hypertensive arteriopathy or iCAA, and without hereditary CAA.

**Results:**

Between November 2023 and January 2024, 2/35 (6%, 95% confidence interval 2%–19%) outpatient clinic patients had a history of RBC transfusion and none had a high likelihood medical history. The cases presented at age 47 and 57 and had already developed severe CAA.

**Conclusions:**

Red blood cell transfusion might be a possible mechanism for iCAA; however, further prospective data collection and experimental evidence concerning blood transmission of amyloid‐β are needed.

## BACKGROUND

Iatrogenic cerebral amyloid angiopathy (iCAA) is thought to be caused by amyloid‐β transmission through medical procedures. Since its recognition >70 cases have been published, mostly linked to using cadaveric human materials derived from the central nervous system [1, 2, 3].

In 2020, a large international consortium called for surveillance of blood transfusions as a possible transmission source of amyloid‐β [4]. In a recent study, recipients of red blood cell (RBC) transfusions from donors who developed multiple spontaneous intracerebral hemorrhages (ICHs) had increased risk of ICH themselves [5]. This was hypothesized to be related to transmission of CAA.

The first two patients who had RBC transfusions as an infant and presented with CAA decades later are presented.

## METHODS

Cases were identified by systematically and prospectively asking all patients who visited our outpatient CAA clinic between November 2023 and January 2024 about a history of, or with a high likelihood for, receiving RBC transfusions (e.g., hemolytic disease or high energy trauma with massive hemorrhage) [6]. Only patients diagnosed with sporadic CAA (according to the Boston criteria 2.0), non‐hereditary young‐onset CAA (age at presentation <55 years), CAA with concomitant hypertensive arteriopathy (mixed CAA‐HTA) or iCAA were considered [7]. Patients with RBC at any time were recorded, but only those with (possible) exposure before first CAA‐related symptoms were considered for reporting.

This study was performed at the Leiden University Medical Centre (LUMC), a CAA referral center in the Netherlands. Written informed consent was obtained from both reported cases. The LUMC Medical Ethics Committee approved this study, concluding that it does not fall under the medical research on human subjects act (non‐WMO; protocol G19.077). This report follows the CARE and STROBE reporting guidelines.

## RESULTS

Thirty‐five patients (age, median 68 years, range 43–83 years) from the outpatient clinic were screened between November 2023 and January 2024: 28 with sporadic CAA, three cadaveric dura confirmed possible iCAA and four with mixed CAA‐HTA. Two (6%; 95% confidence interval 2%–19%) patients who had a history of RBC transfusions were identified.

### Case 1

**FIGURE 1:**
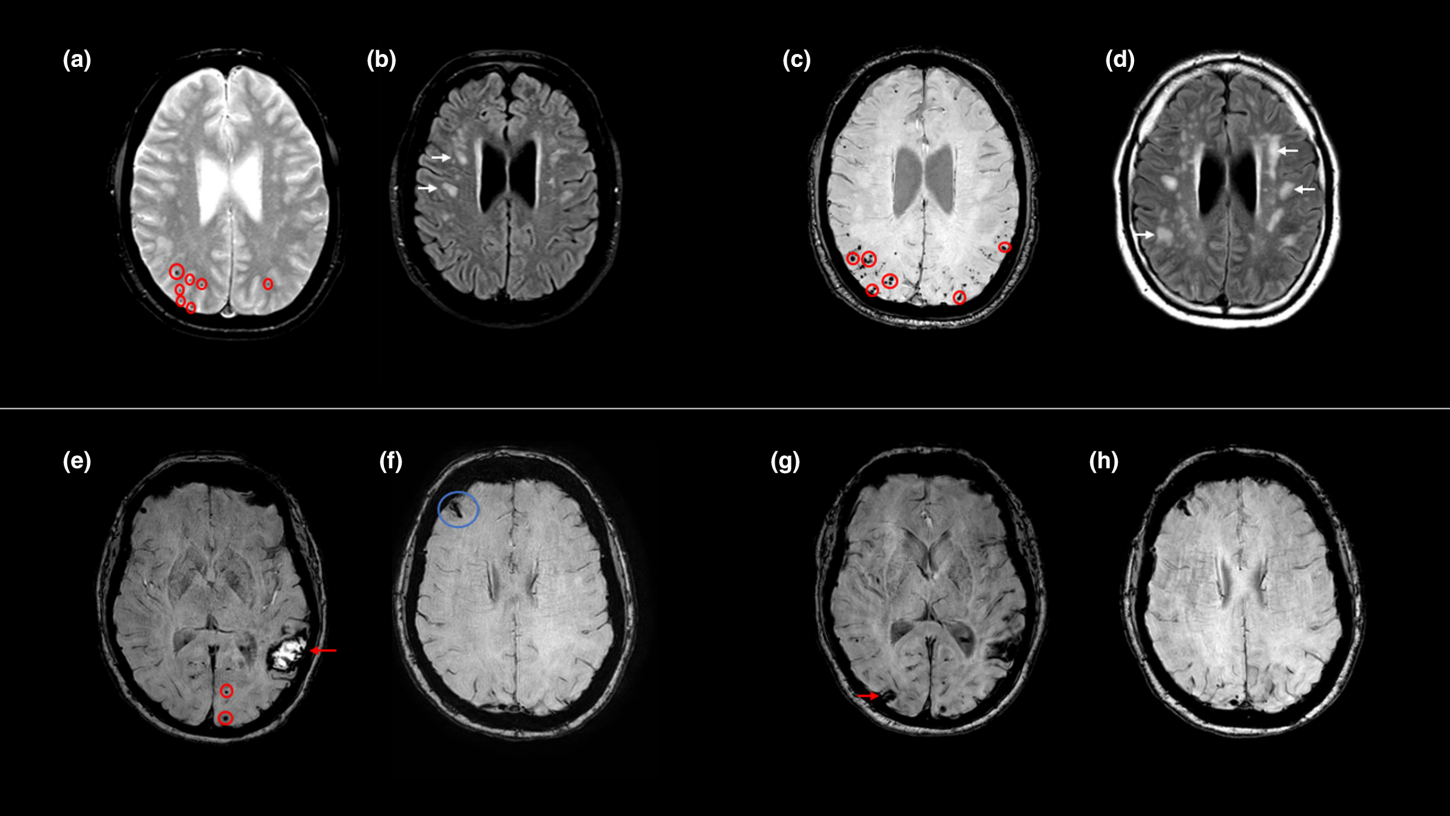
Radiological findings of both cases. Case 1: First MRI (1.5 T) showed >20 cerebral microbleeds (CMBs) ((a) red circles; T2 hemo‐sensitive), predominantly in the parietal, occipital and temporal lobes. There were focal white matter hyperintensities (WMHs) ((b) white arrows; T2 fluid attenuated inversion recovery, FLAIR) with beginning confluence and enlarged perivascular spaces in the centrum semi‐ovale (CSO‐EPVS; not shown; 21–40 visible; T2 turbo spin echo), in the absence of cortical superficial siderosis (cSS), previous ICH or deep hemorrhagic markers. Follow‐up MRI (3 T) after 4 years showed >100 CMBs ((c) susceptibility weighted imaging, SWI) and multispot pattern WMHs ((d) FLAIR). Case 2: First MRI and follow‐up after 2 months (performed due to temporary hemiparesis and hemihypesthesia) showed a recent left temporal ICH ((e) red arrow; SWI) with multiple lobar CMBs (red circles), right frontal focal cSS ((f) blue circle), multiple WMH spots and CSO‐EPVS (1–10 visible; both not shown). Repeat MRI 1 month later, performed due to headache and impaired vision, showed a new right parietal ICH and multispot pattern WMH (not shown). MRI (3 T) at 9 months also showed this ICH (g) and a new left parietal ICH (not shown) without further progression of cSS (h), WMHs or CSO‐EPVS.

The first case concerns a male patient who was known to have well‐regulated generalized epilepsy, who presented in 2019 at age 47 due to an episode with loss of strength in the legs, after which he fell down the stairs and had limb‐jerking of the right arm with a loss of consciousness. An electroencephalogram showed epileptiform activity, after which his anti‐epileptic drugs were altered. Magnetic resonance imaging (MRI) performed elsewhere showed radiological evidence of probable CAA (Figure 1).

The patient did not have a history with neurosurgical intervention but he did have RBC transfusions due to rhesus factor mismatch shortly after birth in 1972 (written documentation obtained).

There was no positive family history for dementia or ICH. Because of his young age, hereditary variants of CAA and Alzheimer's disease (AD) were excluded (genes tested: *APP*, *NOTCH3*, *TREX1*, *HTRA1*, *ABCD1*, *AUH*, *CBS*, *CLCN2*, *COL4A1*, *COL4A2*, *CSF1R*, *CST3*, *CYP27A1*, *CSTA*, *DARS2*, *GBE1*, *GFAP*, *GLA*, *GSN*, *ITM2B*, *LMNB1*, *MMACHC*, *TREM2*, *TTR*, *TYMP*, *TYROBP*). Lumbar puncture, biopsy and amyloid positron emission tomography were not performed. Clinically, the patient remained stable without new complaints throughout a 4‐year follow‐up period, but radiological progression was observed (Figure 1).

### Case 2

The second case concerns a 57‐year‐old female patient who presented at the end of 2022 due to sudden onset headache with difficulty in recognizing familiar faces and increasing confusion. She has a history with migraine and severe headaches and did not seek medical attention. The next morning she woke up with new dysarthria, word‐finding difficulties and right‐hand weakness. Computed tomography imaging showed a left temporal ICH. MRI several days later additionally revealed multiple lobar cerebral and cerebellar microbleeds and focal cortical superficial siderosis (Figure 1).

There was no family history of ICH or dementia, and hereditary variants of CAA and AD were excluded (same gene panel as above). Lumbar puncture, biopsy and amyloid positron emission tomography were not performed. Other than migraine and her receiving an RBC transfusion as an infant because of rhesus factor mismatch, her medical history was unremarkable. Written documentation of the RBC transfusions could not be obtained (medical records destroyed; correspondence available). The patient also has a sister 5 years older who received RBC transfusions but does not have any symptoms.

Two months after the first presentation, the patient had a temporary left hemiparesis and hemihypesthesia of which the origin is unclear. This presentation might have been due to a transient focal neurological episode, although no corresponding cortical superficial siderosis or convexity subarachnoid hemorrhage was observed on MRI. One month later, she had complaints of a new headache and impaired vision. Repeat MRI showed a new ICH of the right parietal lobe. Six months later, further progression was visible on MRI (Figure 1).

## DISCUSSION

Two cases were identified with CAA decades after receiving RBC transfusions. Both cases were relatively young—one too young to fulfil the Boston criteria 2.0—and had already developed severe CAA. In the light of the recent blood transfusions paper, these cases create a starting point for further clinically investing the frequency of a history with RBC transfusions in patients with CAA, such that a possible overrepresentation might be recognized in the years to come [5].

Recognition of new modes of transmission of iCAA is important, as there may be implications for public health. For example, should RBC transfusions later become recognized to cause CAA, this could have implications for patients with CAA donating blood. There is some pathophysiological basis for transmission of amyloid‐β through blood transfusions and current evidence regarding transmissibility of amyloid‐β has been extensively discussed [8].

First, in some animal studies CAA was induced by peripheral (intraperitoneal or intravenous) injection of amyloid‐β [8, 9]. It is noted, however, that the required concentration for this induction was estimated to be 1000 times higher than in intracerebral inoculations [9]. Secondly, in the cadaveric‐dura‐related iCAA literature, several procedures involved non‐cranial surgeries, such as cardiac surgery, external carotid embolization, resection of nasopharyngeal hemangioma or spina bifida repairs [1, 2, 10, 11]. Thirdly, blood products are an established mode of transmission in other protein‐mediated transmissible diseases, such as variant Creutzfeldt–Jakob disease [12]. Blood as a spreading route for amyloid‐β might therefore not be an unreasonable hypothesis.

The proportion of RBC transfusions in our population (6%; 95% confidence interval 2%–19%) might be contrasted by crudely calculating the childhood transfusion rate in the Netherlands. In 1996–2006, 7 million RBC products were issued (4.4% for patients aged <20 years; mean 8.3 products per recipient), and the average population was 15.9 million. Therefore, 0.23% of Dutch citizens might have been exposed during childhood [13, 14]. This is similar to the low lifetime prevalence of receiving RBC transfusions (yearly rate 27/1000 persons per year in the Netherlands). Simultaneously, ≤1% of donors might later develop ICH, which renders a low absolute risk of potential exposure to exogenous amyloid‐β after RBC transfusions [5, 15]. Moreover, a large Scandinavian registry study did not observe evidence for transmission of neurodegenerative diseases including AD (another amyloid‐β mediated disease), in line with other research on the topic [16, 17]. It should therefore be stressed that the current state of the evidence is too meagre to draw conclusions for transfusion‐related transmissibility of CAA in clinical practice at this moment and that, for now, the most likely diagnosis in both our cases remains young‐onset and sporadic CAA.

A strength of our report is that it provides a starting point for exploring this new possible cause of iCAA. By prospectively asking all patients at our outpatient clinic, it was possible to obtain data as informative as currently feasible.

The following limitations are noted. Unfortunately, it was not possible to ascertain clinical information about the donor (including ICH or CAA later in life) or to obtain saved plasma samples due to donor‐related privacy jurisdiction in the Netherlands. This limitation might be overcome in larger registry‐based studies aimed at replication of the findings in the recent blood transfusions publication that linked ICH in donors and recipients [5]. Further, documentation about the number of transfusions in our patients was missing. However, because research on iCAA concerns exposures of decades ago, it is expected that in many future cases this information will remain missing. The history of RBC transfusions was obtained during medical history taking, specifically asking for RBC transfusions. Patients were not instructed to ask their parents about a history of transfusions during childhood. Also, patients might have forgotten that they had transfusions a long time ago. This might have caused underestimation due to recall bias, which might be minimized in future prospective studies by instructing included patients to validate their medical history with an informant (e.g., parents or spouse). Another limitation is our low sample size, because enquiries about RBC transfusions were only started after the recent blood transfusions publication [5]. This might be further increased at a later time. Finally, a representative control group could not be prospectively sampled to assess exposure in the source population, and controls from previous studies were anticipated to provide biased estimates. Once more cases become recognized, a standardized mortality ratio or odds ratio for developing CAA after RBC transfusion might be calculated.

For now, the next step for clinicians is systematically to ask patients with CAA about a history with RBC transfusions, so this possible exposure mechanism can be further elucidated. At the same time, the ball is up to experimental researchers to investigate transfusion‐related development or acceleration of CAA.

## AUTHOR CONTRIBUTIONS

**K. Kaushik:** Writing – original draft; investigation; methodology; data curation; formal analysis; project administration; visualization. **M. J. H. Wermer:** Funding acquisition; investigation; methodology; writing – review and editing; supervision; visualization; resources. **E. S. van Etten:** Conceptualization; investigation; methodology; writing – review and editing; supervision; project administration; visualization.

## FUNDING INFORMATION

M.J.H. Wermer reports independent support from the Netherlands Organization for Scientific Research (NWO VIDI grant 9171337, Aspasia grant), the Dutch Heart Foundation (Clinical Established Investigator grant 2016T86) and the Dutch Brain Foundation.

## CONFLICT OF INTEREST STATEMENT

All other authors report no disclosures.

## Data Availability

The data that support the findings of this study are available on request from the corresponding author. The data are not publicly available due to privacy or ethical restrictions.
